# Risk allelic load in Th2 and Th3 cytokines genes as biomarker of susceptibility to HPV-16 positive cervical cancer: a case control study

**DOI:** 10.1186/s12885-016-2364-4

**Published:** 2016-05-24

**Authors:** K. Torres-Poveda, A. I. Burguete-García, M. Bahena-Román, R. Méndez-Martínez, M. A. Zurita-Díaz, G. López-Estrada, K. Delgado-Romero, O. Peralta-Zaragoza, V. H. Bermúdez-Morales, D. Cantú, A. García-Carrancá, V. Madrid-Marina

**Affiliations:** Dirección de Infecciones Crónicas y Cáncer. Centro de Investigación sobre Enfermedades Infecciosas, Instituto Nacional de Salud Pública (INSP), (Chronic Infectious Diseases and Cancer Division. Center for Research on Infectious Diseases. National Institute of Public Health Mexico), Av. Universidad 655, Santa María Ahuacatitlán, Cuernavaca, C.P.62100 Morelos Mexico; CONACyT Research Fellow-Instituto Nacional de Salud Pública (INSP), Cuernavaca, Morelos Mexico; Division of Basic Research, Instituto Nacional de Cancerología (INCan), SS. Mexico City, Mexico; Private Health Center for Gynecology, Cuernavaca, Morelos Mexico; Centro de Atención para la Salud de la Mujer (CAPASAM), (Center for Women’s Health). Health Services of the State of Morelos, Cuernavaca, Mexico; Division of Clinical Research, Instituto Nacional de Cancerología (INCan), SS. Mexico City, Mexico; Unit of Biomedical Research in Cancer, Instituto Nacional de Cancerología (INCan), SS and Biomedical Research Institute. Universidad Nacional Autónoma de México, Mexico City, Mexico

**Keywords:** Genetic susceptibility profile, Cytokines, Promoter polymorphisms, Serum levels, Cervical neoplasm

## Abstract

**Background:**

Alterations in the host cellular immune response allow persistent infections with High-Risk Human Papillomavirus (HR-HPV) and development of premalignant cervical lesions and cervical cancer (CC). Variations of immunosuppressive cytokine levels in cervix are associated with the natural history of CC. To assess the potential role of genetic host immunity and cytokines serum levels in the risk of developing CC, we conducted a case–control study paired by age.

**Methods:**

Peripheral blood samples from patients with CC (*n* = 200) and hospital controls (*n* = 200), were used to evaluate nine biallelic SNPs of six cytokine genes of the adaptive immune system by allelic discrimination and cytokines serum levels by ELISA.

**Results:**

After analyzing the SNP association by multivariate logistic regression adjusted by age, CC history and smoking history, three Th2 cytokines (IL-4, IL-6 and IL-10) and one Th3 (TGFB1) cytokine were significantly associated with CC. Individuals with at least one copy of the following risk alleles: T of SNP (−590C > T IL-4), C of SNP (−573G > C IL-6), A of SNP (−592C > A IL-10), T of SNP (−819C > T IL-10) and T of SNP (−509C > T TGFB1), had an adjusted odds ratio (OR) of 2.08 (95 % CI 1.475–2.934, *p* = 0.0001), an OR of 1.70 (95 % CI 1.208–2.404, *p* = 0.002), an OR of 1.87 (95 % CI 1.332–2.630, *p* = 0.0001), an OR of 1.67 (95 % CI 1.192–2.353, *p* = 0.003) and an OR of 1.91 (95 % CI 1.354–2.701, *p* = 0.0001), respectively, for CC. The burden of carrying two or more of these risk alleles was found to have an additive effect on the risk of CC (p trend = 0.0001). Finally, the serum levels of Th2 and Th3 cytokines were higher in CC cases than the controls; whereas IFNG levels, a Th1 cytokine, were higher in controls than CC cases.

**Conclusion:**

The significant associations of five SNPs with CC indicate that these polymorphisms are potential candidates for predicting the risk of development of CC, representing a risk allelic load for CC and can be used as a biomarker of susceptibility to this disease.

## Background

Cervical cancer (CC) ranks third as a cause of death among women worldwide, with an estimated overall mortality rate of 15 per 100,000 women [1]. CC was the second most common cause of death in Mexican women in 2011 (10.4 %) [2]. The immune system plays key roles during HPV-associated carcinogenesis, as HPV clearance is determined by specific immunological reactions [3]. Thus, CC seems to be due in part to a failure of the immune system, which is unable to eliminate persistent HPV infections and virus-transformed cells [4].

A local predominance of T helper 2 (Th2) cytokine profile expression (IL-4, IL-6, IL-10) and an impaired cellular immune response induced by immune suppressor cytokines, such as IL-10 and Transforming Growth Factor Beta 1 (TGFB1) in association with a diminished Th1 profile, has been demonstrated in patients with CC [5, 6]. Cytokines are key players in the immune system and several polymorphisms identified in their gene promoters may be responsible for variations in expression levels observed between individuals in different diseases [7].

Single nucleotide polymorphisms (SNPs) in IL-4, IL-6, IL-10, TGFB1, Tumor Necrosis Factor-alpha (TNF) and Interferon-gamma (IFNG) genes that cause variations in host immune response may contribute to CC risk. Since the boom of the genomic era, there are have been numerous reports of polymorphisms associated with CC risk; however, very few of the studied polymorphisms in this neoplasm have shown consistent associations across studies and populations. A large number of SNPs have been identified in IL-4, IL-6, IL-10, TGFB1, TNF e IFNG and many of them have been studied regarding their association with CC in different populations, particularly -592C > A, −819C > T and -1082A > G of IL-10, −509C > T TGFB1 and -308G > A of TNF [8].

In this study, we analyzed the association between -590C > T (IL-4), −573G > C (IL-6), −592C > A (IL-10), −819C > T (IL-10), −1082A > G (IL-10), −509C > T (TGFB1), −800G > A (TGFB1), −308G > A (TNF) and -1615C > T (IFNG) gene promoter polymorphisms and the risk for CC in Mexican women. These SNPs were selected based on two criteria: validated SNPs for frequency or for utilization in the HAPmap Project and SNPs in the promoter region with potential role in transcriptional regulation of each cytokine evaluated. Furthermore, we determined whether there are variations in serum protein levels of these cytokines according to diagnostic and allelic variation of each SNP.

## Methods

### Study design and population

A clinic-based case–control study matched by age (±5 years) 1:1, (*n* = 200 per group) was conducted at the National Cancer Institute’s (INCan) Gynecology Service in Mexico City between September 2010 and December 2011. Cases with cervical squamous cell carcinoma confirmed by two pathologists were included, and women with a negative Papanicolaou study and a normal colposcopy were selected as controls and matched to the cases by age (±5 years). All participants were HPV 16-positive, since this was an inclusion criterion. The analyzed population was Mexican mestizo with a history of three previous generations being born in Mexico and with a residency period of > 1 year in the study area. To eliminate any selection bias, the authors screened CC cases and controls to ensure that they had never been diagnosed with chronic inflammatory diseases. The Bioethics and Research Committees at INCan (reference INCan/CC/326/10CB/609) and at the Instituto Nacional de Salud Pública (INSP, reference CI814 and CI1289 No. 1624) approved the study, which was carried out according to the Helsinki Declaration. All participants signed an informed consent to participate in this study, agreeing also to the subsequent publication of the results. Each subject was interviewed regarding lifestyle and socio-demographic and hormonal factors known to be associated with an increased risk of CC.

### Specimen collection and sample processing

Cervical epithelial cell scrapings from the controls and fresh cell biopsies from the women diagnosed with CC were used for this study. Peripheral blood mononuclear cells (PBMC) from all subjects were obtained by Ficoll-hypaque density gradients (Hystopaque, Sigma Chemical Co). Genomic DNA was extracted from PBMC using the Genomic DNA Purification Kit (Fermentas Life Sciences, Lithuania) and from cervical epithelial scrapings and biopsies previously digested with proteinase K. DNA concentration and purity were evaluated with a Thermo Scientific NanoDropTM 1000 Spectrophotometer (260/280) and the integrity of the DNA was determined by electrophoresis in agarose gels at 0.8 %.

Cervical epithelial scrapings and biopsy specimens were tested for HPV by PCR amplification, using consensus primers MY09/MY11, LIC1/LIC2, and GP5/GP6. GAPDH (250 bp) was used as an internal control for DNA quality; SiHa was used as a positive control, and deionized H2O as a negative control. The purified DNA band was sequenced using the Sanger method. After analyzing the sequences by BLAST, only HPV 16-positive women were included in this study. Serum protein levels of IL-4, IL-6, IL-10, TGFB1, TNF, and IFNG were determined using the Human high sensitivity ELISA kit (Abcam, Cambridge UK). All assays were done by duplicate and the final concentration (picograms per millilitre [pg/ml]) and TGFB1 (nanograms per millilitre [ng/ml]) of each cytokine corresponded to the average of the duplicate readings.

### Genotyping

The SNPs selection criteria were as follows: 1) Validated SNPs for frequency or for utilization in the HAPmap Project; 2) SNPs in the promoter region with potential role in the transcriptional regulation of each cytokine evaluated (the Ensemble program was used for this selection); 3) SNPs in IL-4, IL-6, IL-10, TGFB1, TNF and IFNG promoter, located in the binding sites of the transcription factors that potentially influence the transcriptional activity, reported in the following database: SNPper.URL: http//snpper.chip.org. Nine SNPs in the promoter region were genotyped using PCR with TaqMan fluorogenic probes in the ViiA™ 7 (Applied Biosystems, Foster City, CA, USA), (−590C > T (IL-4) rs2243250, −573G > C (IL-6) rs1800796, −592C > A (IL-10) rs1800872, −819C > T (IL-10) rs1800871, −1082A > G (IL-10) rs1800896, −509C > T (TGFB1) rs1800469, −800G > A (TGFB1) rs1800468, −308G > A (TNF) rs1800629 and -1615C > T (IFNG) rs2069705). All tests were performed in duplicate. The alleles were assigned using the ViiA™ 7 software (Applied Biosystems). A call rate of 0.99 for controls and CC was used for quality control. When the call rate was less than 0.99, DNA was reextracted and a new genotyping was performed (only six samples required this procedure).

### Statistical analysis

Reproductive factors and sexual lifestyle were evaluated by multinomial logistic regression models adjusted for age. The Hardy-Weinberg equilibrium for each SNP was assessed using the allelic frequencies of the control group. Genotype-specific and allele risks were estimated as odds ratios (OR) with associated 95 % confidence intervals (CI). All *p*-values were based on a two-sided hypothesis test using logistic regression analyses in the three inheritance models and adjusting by potential confounders (age, CC history and smoking history). The Bonferroni method was used to correct the multiple comparisons (*α* = 0.05/9 = 0.0055). The effect of having one or more risk-associated alleles with CC was evaluated using multiple logistic regressions. All possible 2-way interactions among SNPs and between SNPs and serum level of IL-4, IL-6, IL-10, TGFB1, TNF e IFNG were tested by multivariate logistic models. Tertiles for the serum levels of each cytokine according to the respective observed distribution in the HPV-positive control group were created to evaluate their association with CC by multinomial logistic regression models. The mean differences of the serum levels of cytokines between CC and controls stratified by genotypes were assessed by linear regression models adjusted by age, CC history and smoking history.

Stratification by ethnicity that could potentially confound genetic analyses was avoided by confining our analysis to the Mexican mestizo population with two previous generations born in Mexico. The power calculation for each of the analyzed SNPs was evaluated taking into account the value of n fixed for the study and the minor allele frequency for each SNP in the NCL (P1), with an expected OR of 1.2, 1.5, 2, 2.5 and 3. The established alpha value was 0.05. The P2 calculation was performed using the following formula: P1*OR/(1-P1) + (P1*OR). The statistic power obtained in the regression analysis for each SNP was as follows: −590 C > T of IL-4 (0.99); −573 G > C of IL-6 (0.92); −592 C > A (0.98), −819 C > T (0.91) and −1082 A > G (0.14) of IL-10; −509 C > T of TGFB1 (0.98); −308 G > A of TNF (0.28) and −1615 C > T of IFNG (0.99). All the statistical analyses were performed in the STATA program, version 13.0 (StataCorp, Collage Station, TX, EUA).

## Results

The analyses confirmed that known reproductive and sexual lifestyle risk factors for CC were statistically different between study groups. As expected, we found a significant positive association of CC diagnosis with age at first intercourse, parity, number of lifetime sexual partners, cancer family history and smoking history. The study did not find the history of previous Sexually Transmitted Diseases (STDs) and use of contraceptive methods to be risk factors for CC in this population (Table 1). Given the best score in the goodness of fit tests performed for all the logistic models evaluated, we carried out further multinomial logistic regression analysis, adjusting for cancer family history, smoking history and age. We observed no significant deviations from HWE among controls in any of the genotyped SNPs. The SNP -800G > A (TGFB1) rs1800468, was found not to be polymorphic with a minor allele frequency (MAF) of <1 % (Table 2).

**Table 1:** Analysis of the reproductive and sexual life style conventional risk factors for cervical cancer in study population

Sociodemographic characteristics	*n* (%) Controls/*n* (%) CC	OR^c^ (95 % CI)	
	*n* = 200/200	Cervical cancer (CC)	*p* value^a^
Age of menarche (years)			
<12	82 (41)/76 (38)	1 (reference)	
≥12	118 (56)/124 (62)	0.76 (0.47–1.22)	0.26
Age at first intercourse (years)			
≥18	114 (57)/50 (25)	1 (reference)	
<18	86 (43)/150 (75)	**5.64 (3.32–9.56)** ^b^	**0.0001**
Parity			
≤3	177 (88.5)/71 (35.5)	1 (reference)	
>3	23 (11.5)/129 (64.5)	**7.41 (4.23–12.98)** ^b^	**0.0001**
Number of lifetime sexual partners			
<3	169 (84.5)/148 (74)	1 (reference)	
4 a 9	28 (14)/50 (25)	**2.27 (1.24–4.15)** ^b^	**0.008**
>10	3 (1.5)/2 (1)	2.79 (0.42–18.41)	0.28
Contraceptive method			
None	28 (14)/111 (55.5)	1 (reference)	
Non hormonals	88 (44)/56 (28)	**0.25 (0.139–0.458)**	**0.0001**
Hormonal methods 6 months-5 years	84 (42)/33 (16.5)	**0.15 (0.08–0.29)** ^b^	**0.0001**
History of previous STD			
None	109 (54.5)/144 (72)	1 (reference)	
Herpes	5 (2.5)/2 (1)	0.4 (0.065–2.44)	0.32
Chlamydia	1 (0.5)/1 (0.5)	0.46 (0.028–7.50)	0.58
Candidiasis	33 (16.5)/1 (0.5)	**0.02 (0.003–0.235)**	**0.001**
Vaginosis	33 (16.5)/49 (24.5)	1.32 (0.73–2.39)	0.34
HPV	19 (9.5)/3 (1.5)	**0.18 (0.04–0.69)**	**0.01**
Cancer family history			
No	176 (88)/158 (79)	1 (reference)	
Yes	24 (12)/42 (21)	**2.19 (1.15–4.16)** ^b^	**0.01**
Smoking history			
No	155 (77.5)/125 (62.5)	1	
Yes	45 (22.5)/75 (37.5)	**2.15 (1.28–3.63)** ^b^	**0.004**

Bold text denotes significant *p* values (*p* < 0.05)

^a^ *p* value for Kruskal-Wallis test

^b^Statistically significant *p* values for trend (*p* < 0.05)

^c^Odds Ratio adjusted by age

**Table 2:** Association analysis of SNP in promoter of IL-4, IL-6, IL-10, TGFβ-1, TNF-α, IFN-γ with CC

Polymorphism	*n* (%) CC/(%) controls (*n* = 200/200)	OR^c^ (95 % CI)	*p* Value*
IL-4 -590C > T (rs2243250)			
Codominant model			
C/C	32 (16)/60 (30)	1	
C/T	87 (43.5)/102 (51)	1.50 (0.804–2.807)	0.2
T/T	81 (40.5)/38 (19)	**4.36 (2.166–8.803)** ^b^	**0.0001**
Dominant model			
C/C	32 (16)/60 (30)	1	
C/T + T/T	168 (84)/140 (70)	**2.22 (1.238–3.987)**	**0.007**
Recessive model			
C/C + C/T	119 (59.5)/162 (81)	1	
T/T	81 (40.5)/38 (19)	**3.3 (1.909–5.707)**	**0.0001**
Alleles			
C	151 (37.7)/222 (56)	1	
T	249 (62.25)/178 (44)	**2.08 (1.475–2.934)**	**0.0001**
p HWE^a^			0.6
IL-6 -573G > C (rs1800796)			
Codominant model			
G/G	54 (27)/89 (44.5)	1	
G/C	85 (42.5)/91 (45.5)	1.47 (0.875–2.499)	0.14
C/C	61 (30.5)/20 (10)	**3.2 (1.572–6.535)** ^b^	**0.001**
Dominant model			
G/G	54 (27)/89 (44.5)	1	
G/C + C/C	146 (73)/111 (55.5)	1.82 (1.111–2.983)	0.017
Recessive model			
G/G + G/C	139 (69.5)/180 (90)	1	
C/C	61 (30.5)/20 (10)	**2.58 (1.348–4.934)**	**0.004**
Alleles			
C	193 (48.25)/269 (67.25)	1	
T	207 (51.75)/131 (32.75)	**1.70 (1.208–2.404)**	**0.002**
p HWE^a^			0.72
IL-10 -592C > A (rs1800872)			
Codominant model			
C/C	44 (22)/85 (42.5)	1	
C/A	98 (49)/85 (42.5)	2.08 (1.190–3.656)	0.01
A/A	58 (29)/30 (15)	**3.28 (1.660–6.508)** ^b^	**0.001**
Dominant model			
C/C	44 (22)/85 (42.5)	1	
C/A + A/A	156 (78)/115 (57.5)	**2.40 (1.416–4.072)**	**0.001**
Recessive model			
C/C + C/A	142 (71)/170 (85)	1	
A/A	58 (29)/30 (15)	2.09 (1.166–3.756)	0.013
Alleles			
C	185 (46.37)/255 (63.75)	1	
A	214 (53.63)/145 (36.25)	**1.87 (1.332–2.630)**	**0.0001**
p HWE^a^			0.25
IL-10 -819C > T (rs 1800871)			
Codominant model			
C/C	49 (24.5)/81 (40.5)	1	
C/T	97 (48.5)/85 (42.5)	1.88 (1.078–3.281)	0.02
T/T	54 (27)/34 (17)	**2.6 (1.330–5.116)** ^b^	**0.005**
Dominant model			
C/C	49 (24.5)/81 (40.5)	1	
C/T + T/T	151 (75.5)/119 (59.5)	**2.08 (1.237–3.514)**	**0.006**
Recessive model			
C/C + C/T	146 (73)/166 (83)	1	
T/T	54 (27)/34 (17)	1.77 (0.997–3.174)	0.05
Alleles			
C	195 (48.75)/247 (61.75)	1	
T	205 (51.25)/153 (38.25)	**1.67 (1.192–2.353)**	**0.003**
p HWE^a^			0.15
IL-10 -1082A > G (rs1800896)			
Codominant model			
A/A	121 (60.5)/110 (55)	1	
A/G	70 (35)/78 (39)	0.73 (0.447–1.205)	0.22
G/G	9 (4.5)/12 (6)	0.89 (0.298–2.659)	0.83
Dominant model			
A/A	121 (60.5)/110 (55)	1	
A/G + G/G	79 (39.5)/90 (45)	0.75 (0.466–1.210)	0.24
Recessive model			
A/A + A/G	191 (95.5)/188 (94)	1	
G/G	9 (4.5)/12 (6)	1.004 (0.342–2.944)	0.99
Alleles			
A	312 (78)/298 (74.5)	1	
G	88 (22)/102 (25.5)	0.83 (0.561–1.234)	0.36
p HWE^a^			0.7
TGFB1 -509C > T (rs1800469)			
Codominant model			
C/C	61 (30.5)/80 (40)	1	
C/T	87 (43.5)/96 (48)	1.46 (0.854–2.514)	0.16
T/T	52 (26)/24 (12)	**3.73 (1.842–7.572)** ^b^	**0.0001**
Dominant model			
C/C	61 (30.5)/80 (40)	1	
C/T + T/T	139 (69.5)/120 (60)	1.91 (1.151–3.174)	0.01
Recessive model			
C/C + C/T	148 (74)/176 (88)	1	
T/T	52 (26)/24 (12)	**2.98 (1.593–5.597)**	**0.001**
Alleles			
C	209 (52.25)/256 (64)	1	
T	191 (47.75)/144 (36)	**1.91 (1.354–2.701)**	**0.0001**
p HWE^a^			0.55
TNFα -308G > A (rs 1800629)			
Codominant model			
G/G	164 (82)/161 (80.5)	1	
G/A	26 (13)/36 (18)	0.55 (0.281–1.081)	0.08
A/A	10 (5)/3 (1.5)	1.52 (0.305–7.631)	0.6
Dominant model			
G/G	164 (82)/161 (80.5)	1	
G/A + A/A	36 (18)/39 (19.5)	0.62 (0.332–1.187)	0.15
Recessive model			
G/G + G/A	190 (95)/197 (98.5)	1	
A/A	10 (5)/3 (1.5)	1.74 (0.356–8.519)	0.49
Alleles			
G	354 (88.5)/358 (89.5)	1	
A	46 (11.50)/42 (10.5)	0.74 (0.421–1.305)	0.3
p HWE^a^			0.54
IFN-γ -1615C > T (rs2069705)			
Codominant model			
C/C	143 (71.5)/88 (44)	1	
C/T	51 (25.1)/91 (45.5)	**0.25 (0.149–0.445)**	**0.0001**
T/T	6 (3)/21 (10.5)	**0.11 (0.038–0.351)** ^b^	**0.0001**
Dominant model			
C/C	143 (71.5)/88 (44)	1	
C/T + T/T	57 (28.5)/112 (56)	**0.22 (0.135–0.385)**	**0.0001**
Recessive model			
C/C + C/T	194 (97)/179 (89.5)	1	
T/T	6 (3)/21 (10.5)	**0.19 (0.068–0.581)**	**0.003**
Alleles			
C	337 (84.25)/267 (66.75)	1	
T	63 (15.75)/133 (33.25)	**0.30 (0.203–0.468)**	**0.0001**
p HWE^a^			0.72

Bold text denotes significant *p* values (*p* < 0.006)

*p < =0.006 Multiples comparisons adjustment by Bonferroni method

^a^Hardy-Weinberg Equilibrium in controls

^b^Statistically significant *p* value for trend (*p* < 0.001)

^c^Odds Ratio adjusted by age, CC history and smoking history

We found a highly significant positive association with CC for minor allele homozygotes of -590C > T (IL-4), −573G > C (IL-6), −592C > A (IL-10), −819C > T (IL-10) and -509C > T (TGFB1). Odds ratios and p trend were OR = 4.36, 95 % CI 2.166–8.803 (*p* = 0.0001); OR = 3.2, 95 % CI 1.572–6.535 (*p* = 0.002); OR = 3.28, 95 % CI 1.660–6.508 (*p* = 0.0001); OR = 2.6 95 % CI 1.330–5.116 (*p* = 0.004); and OR = 3.73, 95 % CI 1.842–7.572 (*p* = 0.0001), respectively. For -1615C > T (IFNG) minor allele homozygotes, we also found a significant negative association with CC (OR = 0.11, 95 % CI (0.038–0.351)), accompanied with a significant negative trend (*p* = 0.0001).

**Fig. 1:**
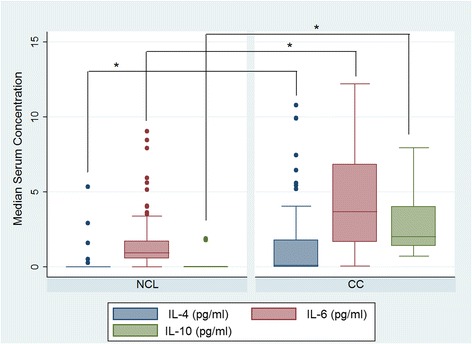
Levels of serum Th2 cytokines in patients with cervical cancer (CC) and controls (NCL). Median serum concentration of IL-4 (pg/ml), IL-6 (pg/ml) and IL-10 (pg/ml). The asterisk represent a statistically significant *p* value for Mann–Whitney test adjusted by multiple comparisons (*p* = 0.00001)

**Fig. 2:**
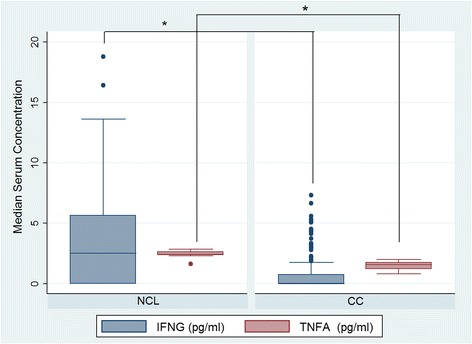
Levels of serum Th1 cytokines in patients with cervical cancer (CC) and controls (NCL). Median serum concentration of IFNG (pg/ml) and TNF (pg/ml). The asterisk represent a statistically significant *p* value for Mann–Whitney test adjusted by multiple comparisons (*p* = 0.00001)

**Fig. 3:**
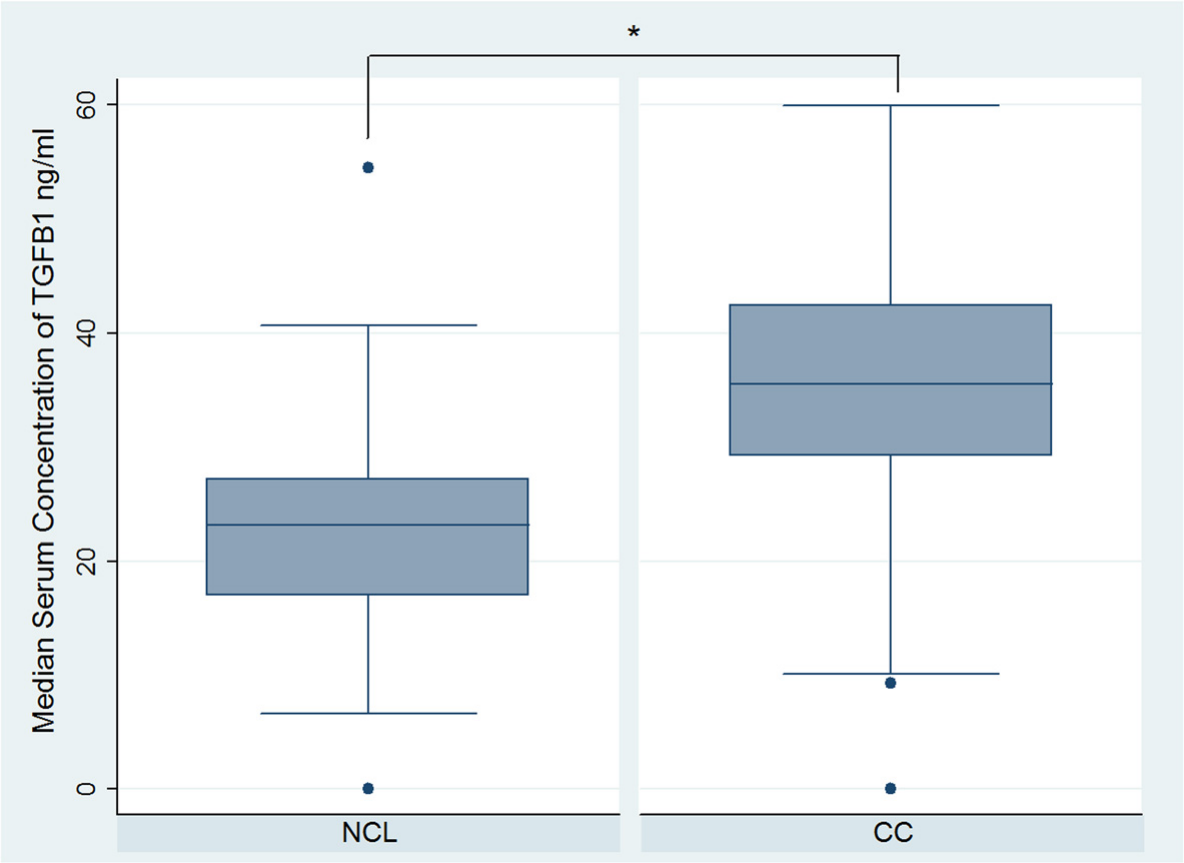
Levels of serum Th3 cytokines in patients with cervical cancer (CC) and controls (NCL). Median serum concentration of TGFB1 (ng/ml). The asterisk represent a statistically significant *p* value for Mann–Whitney test adjusted by multiple comparisons (*p* < 0.00001)

In order to explore whether the evaluated SNPs could act together to increase CC risk, allele load was calculated (Table 3). We indeed found that having two or more risk alleles had a statistically significant association with CC, compared with having no risk alleles, *p* trend = 0.0001. Levels in serum of IL-4, IL-6 and IL-10 were much higher in patients with CC than in HPV-positive control patients (*p* = 0.00001), (Fig. 1). In contrast, IFNG and TNF serum levels were lower than those observed in HPV-positive control patients (*p* = 0.00001), (Fig. 2). Serum levels of TGFB1 were significantly higher in comparison with the other cytokines and the difference between control and CC patients was statistically significant (*p* < 0.00001) (Fig. 3).

**Table 3:** Risk allele load and risk for cervical cancer

SNP’s −590 (IL-4)/-573 (IL-6)/-592 (IL-10)/-819 (IL-10)/-509 (TGFB1)						
Number of alleles associated with CC	*n*			OR^a^	CI 95 %	*P* value
	Total	CC	Controls			
**0**	312	119	193	1	-	
**1**	116	60	56	1.4	0.832–2.386	0.2
**2**	133	69	64	1.75	1.072–2.881	**0.02**
**3**	137	85	52	2.54	1.563–4.159	**0.0001**
**≥4**	99	66	33	2.86	1.608–5.098	**0.0001**
P trend						**0.0001**

*n* = Number of alleles

^a^Odds Ratio adjusted by age, CC history and smoking history

Bold text denotes significant *p* values (*p* < 0.05)

Trend *p* value adjusted by age, CC history and smoking history

We stratified the SNPs of interest by polymorphism genotypes to explore whether they had a relationship with levels of serum cytokines seen in CC patients. Only polymorphisms -592C > A and -819C > T of IL-10, −308G > A (TNF) and -509C > T (TGFB1) showed statistically significant differences in serum cytokines levels between minor allele homozygous and ancestral allele homozygous in CC patients (Table 4). When we evaluated the interaction between each of the SNPs: −590C > T (IL-4), −573G > C (IL-6), −592C > A, −819C > T and -1082A > G (IL-10), −509C > T (TGFB1), −308G > A (TNF) and -1615C > T (IFNG) and respective serum levels, as well as the SNP-SNP interaction, no statistically significant interaction on CC was found (data not shown).

**Table 4:** Estimated mean difference of cytokines serum levels between CC cases and controls stratified by genotypes

Diagnosis	β^a^ coefficient (95 % CI)				
	Polymorphism	Total without stratification	Ancestral allele homozygote	Heterozygote	Minor allele homozygote
	IL-4 -590C > T (rs2243250)		CC	CT	TT
NCL		-	-	-	-
CC		**1.0 (0.696,1.307)**	**0.61 (0.269,0.970)**	1.22 (0.713,1.736)	0.87 (0.214,1.539)
	IL-6 -573G > C (rs1800796)		GG	GC	CC
NCL		-	-	-	-
CC		**2.63 (2.063,3.199)**	**3.12 (2.261,3.994)**	**2.39 (1.567,3.230)**	2.20 (0.367,4.042)
	IL-10 -592C > A (rs1800872)		CC	CA	AA
NCL		-	-	-	-
CC		**2.55 (2.255,2.859)**	**2.27 (1.805,2.737)**	**2.55 (2.063,3.04)**	**2.62 (1.911,3.333)**
	IL-10 -819C > T (rs1800871)		CC	CT	TT
NCL		-	-	-	-
CC		**2.55 (2.255,2.859)**	**2.24 (1.760,2.722)**	**2.45 (1.980,2.927)**	**2.83 (2.111,3.555)**
	IL-10 -1082A > G (rs1800896)		AA	AG	GG
NCL		-	-	-	-
CC		**2.55 (2.255,2.859)**	**2.48 (2.078,2.898)**	**2.77 (2.261,3.291)**	1.13 (0.508,1.758)
	IFN-γ -1615C > T (rs2069705)		CC	CT	TT
NCL		-	-	-	-
CC		**−2.58 (−3.286,-1.891)**	**−2.80 (−3.687,-1.927)**	**−2.61 (−3.929,-1-291)**	−1.7 (−6.097,2.685)
	TNF-α -308G > A (rs 1800629)		GG	GA	AA
NCL		-	-	-	-
CC		**−0.94 (−1.026,-0.860)**	**−0.91 (−1.003,-0.816)**	**−1.08 (−1.30,-0.876)**	**−1.33 (−1.85,-0.813)**
	TGFB1 -509C > T (rs1800469)		CC	CT	TT
NCL		-	-	-	-
CC		**13.4 (11.52,15.47)**	**19.5 (15.85,23.17)**	**11.2 (8.686,13.71)**	**8.7 (2.54,14.98)**

Bold text denotes significant *p* values (*p* < 0.05)

^a^Adjusted by age, CC history and smoking history

Levels of serum cytokines are expressed in pg/ml except TGFB1 (ng/ml)

## Discussion

The main findings of this study were a significant positive association between CC with the SNP’s: −590C > T (IL-4), −573G > C (IL-6), −592C > A (IL10), −819C > T (IL10) and -509C > T (TGFB1) and their serum levels. Although the effect of cytokine gene polymorphisms on CC have been reported in different populations, our results show for the first time a risk allelic load in Th2 and Th3 cytokines genes as a biomarker of susceptibility to CC.

HR-HPV persistent infection is a necessary but not sufficient cause for CC [9]; a combination of several risk factors is required for the disease to develop. The association found in this study for the variables early-age of first sexual intercourse, high number of sexual partners and multiparity, confirms the known association reported in previous epidemiological studies [10].

Genetic predisposing factors may influence the likelihood of, sensitivity to or persistence of HPV infection, as well as the rate of tumor development [11]. Various immune-suppressive states associated with a reduction in cellular immune responses are associated with increasingly severe cervical dysplasia and increased viral shedding, suggesting that a T-helper bias that opposes Th1 responses (ie, Th2 bias) might predispose to chronic infection [12–15].

To our knowledge, this is the first study to show that the IL-4 SNP rs2243250 is significantly associated with the risk of CC. IL-4 is an anti-inflammatory cytokine and the T allele of the SNP -590C > T has been associated with increased transcriptional activity in vitro [16]. The exact mechanism for this increment is not known. However, since the SNP is located within 5′UTR of the gene, it may be possible that alterations in this gen could be influencing in its transcription and/or mRNA stabilization [17]. The IL-4 -590 SNP is a transition (C → T) that has been associated with oral cancer and is a suitable genetic marker for screening for this condition [17]. Increased plasma concentrations of IL-4 can influence the immune status of an affected individual through several mechanisms and result in many phenotypes beyond the scope of the immune system [18].

Likewise, our results showed that the genotype C/C of the IL-6 SNP -573G > C (rs1800796, previously denoted as -572G > C), was significantly associated with the risk of CC when compared with the heterozygous genotype GC. The IL-6 -573 SNP is a transversion (G → C) that was reported as a genetic risk factor of lung cancer risk in Singaporean Chinese non-smoking females [19]. IL-6 is a multifunctional cytokine that can regulate immune and inflammatory responses. In CC patients, high expression of IL-6 correlate with a promoting effect in tumor cell growth by autocrine and/or paracrine processes [20]. Accumulated data of over-expression of IL-6 mRNA and protein in CC cells have showed that the IL-6 protein played important roles in CC development [12, 21, 22]. High levels of IL-6 are associated with the severity of disease and may promote tumor angiogenesis and cancer, which could be genetically determined at an individual level [23]. Clinical studies in CC patients report that elevated IL-6 serum levels are associated with poor prognosis [24]. Variations in the IL-6 gene have been found to be associated with the plasma levels of the protein [20] and functional relevance has been ascribed to several IL-6 variants located in the promoter region, including -573G > C [25]. However, in this study the concentrations of angiogenic cytokines, such as IL-6, do not vary with genotype. This result is similar to other study which explored whether polymorphisms in angiogenic cytokine genes may affect the levels of cytokines and which reported no variation [26].

With respect to IL-10 cytokine gene polymorphisms it has been reported that several important polymorphic sites in the IL-10 gene, including three in the promoter region (−1082 A > G, −819 C > T, −592 C > A) may influence the transcription of IL-10 messenger RNA and the expression of IL-10 in vitro [27]. Several studies have investigated the possible role of IL-10 cytokine gene polymorphisms in CC [28]. The current study has shown that individuals homozygous for the A-allele of the −592 SNP are at three time’s greater odds of having CC as compared to controls. The −592 SNP rs1800872 is a transversion (C → A) that is located in the IL-10 promoter in a region with negative enhancer activity and is associated with loss of this activity [29], between putative consensus binding sequences for Sp1 and a sequence with similarity to that recognized by members of the ETS family proteins. Steinke et al., have demonstrated that the C to A nucleotide exchange results in increased IL-10 gene promoter activity and that the C variant of this SNP is a repressor element [29]. A previous study carried out in 695 CC patients, 115 family-based patients and 586 unrelated controls, in Caucasian population, revealed an increased risk CC for individuals heterozygous for the A-allele of this SNP [30]. Like our study, two recent meta-analyses reported this SNP as a risk factor for developing CC, especially for Asians [28, 31].

Only one other study has investigated the IL-10 SNP -819C > T rs1800871 and CC. In this study the CT + TT genotype combination and T allele, was slightly higher in 150 CC cases as compared with 162 age- and ethnically-matched cervical cytology negative healthy controls. However, contrary to our investigation, the study did not show statistical significance [32]. At this time, a series of seven molecular epidemiological studies and meta-analyses have investigated the association between the IL-10 -819 SNP, a transition (C → T) and the susceptibility to different cancer types among different populations [33]. Nevertheless, the results from these studies are inconsistent. Most studies investigating this SNP have focused on gastric cancer and some have found a reduced risk of gastric cancer among Asians but not among Caucasians [34]. The mechanism of the influence of IL-10-819 SNP on carcinogenesis is still unknown [33].

The IL-10 -1082 SNP rs1800896 is a transition (A → G) and is the most extensively studied polymorphism in the IL-10 gene in cancer susceptibility [35]. Studies link this SNP to high/low IL-10 producer status. Functional analyses have shown that the −1082 region contains a putative ETS-like transcription factor-binding site and that nuclear factors from a monocyte cell line bind to this region. Transient transfection studies in an Epstein-Barr virus-transformed B cell line indicated that the −1082 A allele confers a two fold increase in transcriptional activity of the IL-10 promoter compared to the G allele [36]. This polymorphism has been associated with CC risk in populations from Zimbabwe and Japan [30, 37], while studies in The Netherlands, South Africa, Hungary and China, similarly to our study did not find any association [35]. In the Japanese population, IL-10 -1082 genotypes corresponding to high production of interleukin (GA and GG) were significantly associated with CC severity [37]. The discrepancy of reported results among studies may be explained by the frequency of the G allele in healthy controls [30, 35, 37]. A previously meta-analyses reported in the subgroup analysis by ethnicity of the association of IL-10 -1082A allele with decreased CC susceptibility among whites only (A vs G: OR, 0.39; 95 % CI, 0.32–0.47) [28]. As expected, the G allele frequency obtained in this study (0.25) was similar to that reported for Italy (0.37) [38] and Argentina (0.32) [39], showing no relation with CC risk.

On the other hand, the common TGFB1 promoter SNP -509C-T (rs1800469) was associated with CC in our study, for all models of inheritance and analysis alleles. This SNP is a transition (C → T) linked to a nearly twofold difference in plasma levels among individuals and with risk, progression, and outcome of numerous diseases [40]. This association with the pathogenesis of certain TGFB1-related diseases can be due to transcriptional suppression by AP1 binding to -509C [40].

Only one other study has investigated the SNP -509C > T rs1800469 and CC. In this study 150 CC patients with -509TT had marginal low risk for stage I (*p* = 0.04, OR = 0.95, 95 % CI = 0.91–0.99). However, −509TT genotype of TGFB1 was associated with increased risk of stage II of cancer (*p* = 0.07, OR = 3.13, 95 % CI = 0.87–11.14). In gene-environment interaction, carriers of TGFB1 -509TT genotype with tobacco usage were at higher risk of CC (OR = 3.67, 95 % CI = 0.38–35.1) [41]. The association found in this study is according to previous report where TGF-beta family members play a key role in cellular growth, proliferation, differentiation and angiogenesis of several cancer types [42] and a marginal protection for early stage 1B but risk for stage II of CC of TGFB1 -509 T allele has been reported [41].

Our results indicated that TNF −308 SNP rs1800629, a transition (G → A), had no effect on CC risk in the overall analysis. This polymorphism is the most studied polymorphism in terms of association with CC and severity with conflicting results. Four recent meta-analysis, pooled data from eight, twelve, fifteen and eighteen case–control studies respectively, have suggested that TNF-308G > A polymorphism is associated with an increased risk of CC [43–46]. The analysis stratified by ethnicity, showed that there was a significant association of this polymorphism and increased risk of CC in Asians [47] and in Caucasian and African populations [45]. In our study, the lack of association between TNF −308 G > A polymorphism and CC was consistent with previous reports from African [48, 49] and Caucasian ethnicity [39, 50, 51]. Differences in ethnic compositions; genetic background, sizes of samples, population stratification, or selection of symptoms of disease between analyzed populations could explain these controversial results [52].

Additionally, our results indicate that the variant heterozygote CT and the homozygous TT of SNP -1615C > T of IFNG was associated with a decreased risk of CC. To our knowledge, this is the first study to show that the IFNG SNP rs2069705 was significantly associated with decreased risk of CC. Studies so far have only investigated this SNP in connection with the risk of breast cancer and SLE [53, 54]. Based on the evidence that the T allele is associated with a higher level of IFNG than the C allele the association of this SNP with this type of cancer is predictable, given that at breast cancer diagnosis, inflammatory cytokines are detected at elevated levels [54]. In contrast, a protective effect for the T allele in CC could be related to an increased expression of IFNG and play a role in protection against persistent HPV infection associated to CC development [55].

Nevertheless, a consensus seems to be emerging in that the combined effect of several cytokine SNPs may play a more crucial role in development disease. Thus, in order to explore whether the polymorphisms we examined could act together to increase risk for CC, we combined alleles and tested the effect on risk for the combinations. Individuals carrying two or more of the risk-associated alleles (−590 (IL-4)/-573 (IL-6)/-592 (IL-10)/-819 (IL-10)/-509 (TGFB1) were at increased risk for CC, compared to those with no risk-associated alleles. Although our study did not have the power to examine multiplicative interactions of specific genotypes, the combination of risk-associated alleles produced notably high odds ratios. This exploratory approach supported the idea that combinations of risk-associated alleles have an additive effect on the risk of CC.

In addition, we have found increased serum level of IL-4, IL-6, IL-10 and TGFB1 cytokines among our CC patients. Levels of IFNG were lower in the group of women with CC compared to controls. This is in accordance with previous works indicating a shift towards a T helper 2-type (Th2) cytokine pattern in CC [56] and that increased serum levels of IL-6, IL-10, and TGFB1 are associated with progression of CC during different stages, all of which have the potential to be angiogenic amplifiers [57].

Given that cytokines contribute importantly to each step of cancer development and progression, and that deregulated levels of cytokines and cytokine receptors can be detected in cancer patients locally and systemically [58], this study demonstrated an anti-inflammatory genetic profile, which is associated with increased susceptibility to cervical cancer. However, no statistically significant interactions were obtained between the polymorphisms cytokines of interest and respective serum levels.

IL-4 could possibly be exerting inhibitory effects on the expression and release of proinflammatory cytokines, IL-6 could be inducing prometastatic genes that subsequently lead to proliferation, and prolonged survival of cancer cells, given that the IL-6 serum levels are associated with poor prognosis [24]. Increased serum IL-10 levels could facilitate development of tumors by suppressing the expression of MHC class I and II antigens and preventing tumor antigen presentation to CD8-cytotic T lymphocytes [31]. In addition to its immunosuppressive effects, IL10 can induce transcription of the HPV16 E7 oncoprotein [59]. Additionally, in one of our previous studies, systemic IL-10 mRNA expression level and the IL-10 protein level in serum are significantly higher in cervical lesions compared to women without lesions. We postulate that IL-10 and TGFB1 induce immune system evasion through an immunosuppressive state in the environment of the cervix in women infected with HPV [6].

Additionally, we are aware of a few limitations in our study, some of which cannot be overcame. First, −based in the patients and the control group randomly from the same hospital, we cannot completely rule out the inherent selection bias. Nevertheless, in order to minimize potential biases, we have made a rigorous epidemiological design of study subjects and more statistical adjustments for known risk factors. Second, our sample is medium sized, which may weaken the statistical power of this study. Under the current sample size, we have 99 %, 98 %, 92 % and 91 % power at a 0.05 significance level to detect an OR of 1.2 or higher for −590 IL-4 and −1615 IFNG, for −592 IL-10 and −509 TGFB1, for −573 IL-6 and for −819 IL-10 polymorphisms, respectively. However, well-designed large, prospective studies with detailed information about HPV infection are required to validate our findings and confirm this hypothesis.

## Conclusions

In conclusion, our results suggest an association of three SNP’s Th2 cytokines (IL-4, IL-6 and IL-10) and Th3 (TGFB1) cytokine with CC. Evaluating the association of the burden of risk alleles −590 (IL-4)/-573 (IL-6)/-592 (IL-10)/-819 (IL-10)/-509 (TGFB1) with CC, we found that carrying three or more risk alleles has an additive effect on the risk of CC. We demonstrate a significant difference in the level of serum proteins IL-4, IL-6, IL-10 and TGFB1 in cases compared to controls (*p* < 0.001). Thus, women with CC have an imbalance in the pattern of cytokines Th1/Th2 given systemically by an increase in type II cytokines (IL-4, IL-6 and IL-10, suppressor of the cellular immune response) and reduced in type I cytokine (INFG), allowing HPV persistence and development of lesions and CC. Finally, Significant associations found for SNP’s −590 (IL-4)/- 573 (IL-6)/-592 (IL-10)/-819 (IL-10)/-509 (TGFB1) with CC, indicate that these polymorphisms are potential candidates for predicting the risk of development of CC, representing a risk allelic load for CC and can be used as a biomarker of susceptibility to this disease. These markers could be useful for early prevention programs and be clinically relevant as immunological biomarkers in patients with cervical lesions.

## Abbreviations

CC: cervical cancer; CI: confidence interval; ETS family proteins: E26 transformationspecific family proteins; GAPDH: glyceraldehyde-3-phosphate dehydrogenase; HPV: human papilloma virus; HR-HPV: high-risk human papillomavirus; IFNG: Interferon-gamma; IL-10: interleukin 10; IL-4: interleukin 4; IL-6: interleukin 6; MAF: minor allele frequency; mRNA: messenger RNA; NCL: non-cervical lesions; OR: odds ratio; PBMC: peripheral blood mononuclear cells; PCR: polymerase chain reaction; SNP: single nucleotide polymorphism; STD: sexually transmitted disease; TGFB1: transforming growth factor beta 1; Th: T helper cells; TNF: tumor necrosis factor.
